# Anlotinib combined with temozolomide for brain metastases in small cell lung cancer with early recurrence after whole-brain radiotherapy: a case report

**DOI:** 10.3389/fmed.2026.1824583

**Published:** 2026-05-08

**Authors:** Bolun Jiang, Xiaojie Wang, Teng Zhang, Qilin Liu, Xia Zhang, Bin Zhang

**Affiliations:** 1 The First Hospital of Dalian Medical University, Dalian, Liaoning, China; 2 Department of Gastroenterology, University Town Hospital, Affiliated Hospital of Shandong University of Traditional Chinese Medicine, Jinan, Shandong, China; 3 The Second Hospital of Dalian Medical University, Dalian, Liaoning, China; 4 The Fifth People's Hospital of Dalian, Dalian, Liaoning, China

**Keywords:** anlotinib, brain metastases, magnetic resonance imaging, recurrence, small cell lung carcinoma, temozolomide

## Abstract

**Background:**

Small cell lung cancer (SCLC) is characterised by aggressive biological behaviour and a high propensity for early brain metastasis. Although whole-brain radiotherapy (WBRT) is a standard treatment modality, early intracranial relapse after WBRT remains prevalent and is associated with an exceedingly poor prognosis due to the paucity of effective salvage options.

**Case presentation:**

We report the case of a 50-year-old male patient diagnosed with extensive-stage SCLC with brain and pancreatic metastases. The patient received first-line etoposide plus cisplatin(EP) chemotherapy and WBRT with simultaneous integrated boost, but experienced early intracranial and systemic progression approximately 7 months post-WBRT. Second-line immunotherapy-based treatment also failed to control disease progression. Consequently, the patient was administered oral anlotinib (12 mg, days 1–14) in combination with temozolomide (200 mg, days 1–5) as salvage therapy. The combination achieved durable intracranial disease control, significant systemic tumour regression, and deep tumour marker remission, with a progression-free survival of approximately 6 months.

**Conclusion:**

The combination of anlotinib and temozolomide has been shown to demonstrate promising efficacy as a salvage treatment for patients with early recurrent brain metastases after WBRT in extensive-stage SCLC. This regimen represents a viable therapeutic option with a manageable safety profile and warrants further prospective evaluation in larger clinical trials.

## Introduction

Small cell lung cancer (SCLC) is a highly aggressive malignancy with a pronounced propensity for early and widespread metastasis, particularly to the brain (1). Brain metastases (BMs) have been identified as a major contributing factor to poor prognosis, and their management continues to represent a significant clinical challenge (2, 3). Whole-brain radiotherapy (WBRT) has been a cornerstone of treatment for SCLC-related BMs; however, a substantial proportion of patients experience early intracranial relapse following WBRT, heralding a dismal outcome due to the paucity of effective subsequent therapies (4).

The therapeutic landscape for patients with SCLC who have BMs that have recurred after WBRT is notably constrained (5). Although immune checkpoint inhibitors have transformed the standard treatment approach for extensive-stage SCLC, their effectiveness in managing intracranial disease following WBRT is frequently constrained (6). Furthermore, the blood–brain barrier frequently limits the effectiveness of conventional chemotherapeutic agents against central nervous system involvement, creating an urgent need for novel strategies that combine systemic activity with reliable central nervous system penetration (7, 8).

This case is worthy of note as it demonstrates the potential clinical utility of combining anlotinib, a multi-target tyrosine kinase inhibitor with antiangiogenic properties, with temozolomide, an oral alkylating agent known for its blood–brain barrier penetration, in a patient with early post-WBRT relapse. The dual control of intracranial and extracranial disease, along with sustained progression-free survival, underscores a synergistic treatment approach. This combination addresses the dual challenge of systemic and intracranial disease progression in this high-risk population, offering a viable salvage option worthy of further investigation.

## Case presentation

### Patient information and initial diagnosis

A 50-year-old male patient was admitted on July 5, 2024, having experienced a 2-week history of cough, headache and fatigue. The patient exhibited no significant past medical history, but did have a 30-year smoking history. A chest computed tomography (CT) scan performed on the July 3, 2024 revealed a centrally located mass in the left upper lobe of the lung measuring 3.9 × 3.7 cm, accompanied by a tumour thrombus in the left pulmonary artery. A cranial CT scan conducted on July 8, 2024 revealed the presence of a mass lesion measuring 5.9 × 5.8 cm in the right frontotemporal lobe.

The patient underwent a bronchoscopic biopsy, which was subjected to histopathological and immunohistochemical analysis on July 8, 2024. This confirmed the presence of small cell lung carcinoma, with positivity for CD56, chromogranin A (CgA), and thyroid transcription factor-1 (TTF-1). The Ki-67 proliferation index was determined to be 75%. At the time of diagnosis, the patient exhibited multiple metastatic lesions, including brain metastases and subsequent pancreatic metastasis, as identified on November 21, 2024, in accordance with the criteria for extensive-stage SCLC.

### Initial treatment and disease progression

From July 11 to October 31, 2024, the patient completed 6 cycles of first-line chemotherapy with etoposide plus cisplatin (EP regimen). During chemotherapy, from August 21 to September 3, 2024, the patient received WBRT with simultaneous integrated boost to brain metastases (WBRT PTV 3000 cGy in 10 fractions; lesion PGTV 4000 cGy in 10 fractions).

Approximately 2 months after completion of first-line treatment, disease progression was observed in February 14, 2025, involving the primary lung lesion and pancreatic metastasis. Second-line treatment with tislelizumab combined with irinotecan was initiated; however, the therapeutic response was unsatisfactory.

### Salvage treatment strategy

Following disease progression of the pancreatic tail lesion after second-line treatment, third-line therapy was initiated on April 3, 2025, with a regimen of nab-paclitaxel, carboplatin, and anlotinib. However, systemic treatment was delayed due to grade III thrombocytopenia. This adverse event was managed with oral hetrombopag olamine (2.5 mg once daily), after which the platelet count returned to normal. A critical turning point occurred on May 14, 2025, when brain magnetic resonance imaging (MRI) revealed newly developed metastatic lesions (4.5 × 4.2 cm) in the brainstem and left parietal lobe, indicating intracranial progression approximately 7 months after high-dose brain radiotherapy, accompanied by widespread systemic progression. On May 16, 2025, the regimen was adjusted to oral anlotinib (12 mg on days 1–14) combined with temozolomide (200 mg on days 1–5) in a 21-day cycle. Starting June 12, 2025, nab-paclitaxel was added to the regimen to enhance systemic disease control, forming a combination of nab-paclitaxel (300 mg on day 1), temozolomide (200 mg on days 1–5), and anlotinib (12 mg on days 1–14) in a 21-day cycle, which was continued thereafter.

### Treatment response and outcomes

#### Intracranial lesions

**Figure 1:**
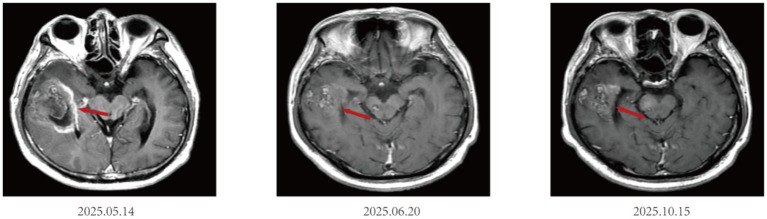
Brain MRI T1WI + C evolution during combination therapy for small cell lung cancer with brain metastases.

After initiation of the combination regimen, brain MRI performed on June 20, 2025, demonstrated effective control of the newly developed metastatic lesion, which measured approximately 4.2 × 2.3 cm. Subsequent follow-up imaging up to October 15, 2025, showed overall stability of multiple brain metastases, with partial lesion shrinkage(4.0*2.3 cm). Representative imaging findings are shown in Figure 1.

#### Systemic lesions

**Figure 2:**
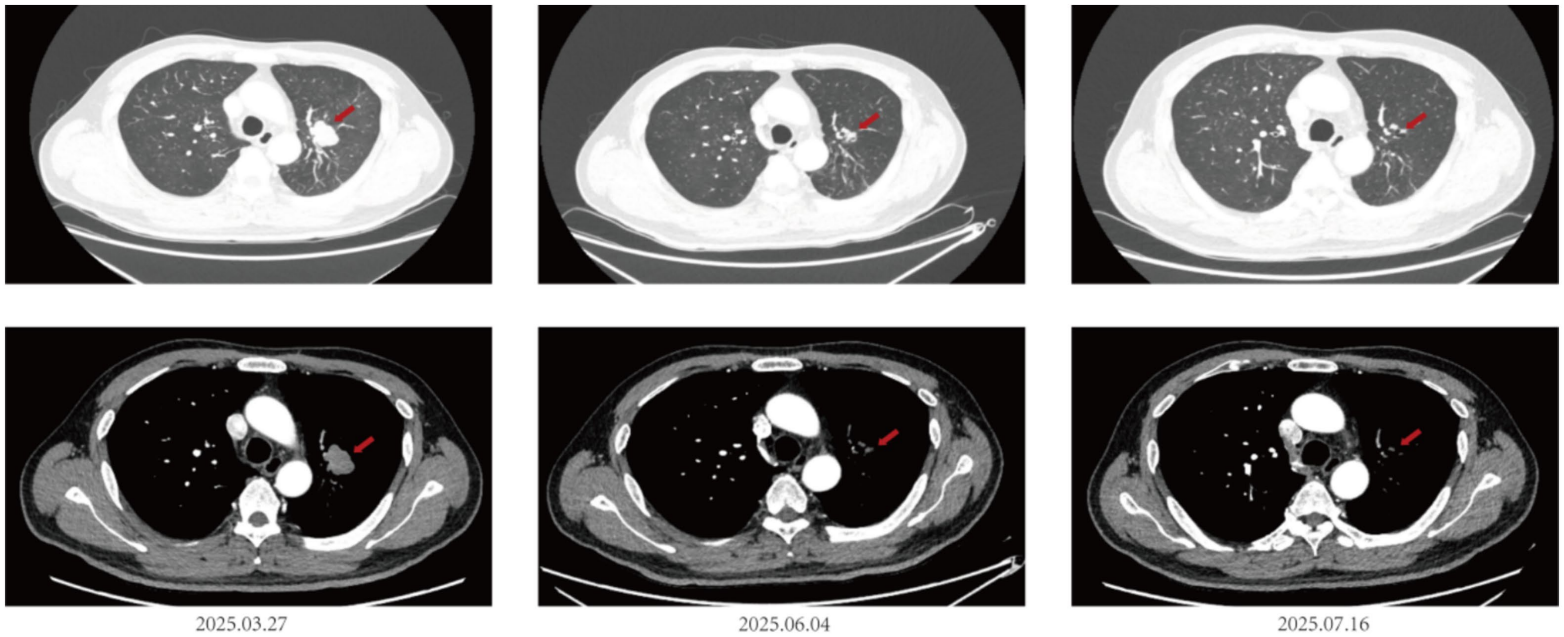
Evolution of chest CT findings during combination therapy in a patient with small cell lung cancer and brain metastases.

**Figure 3:**
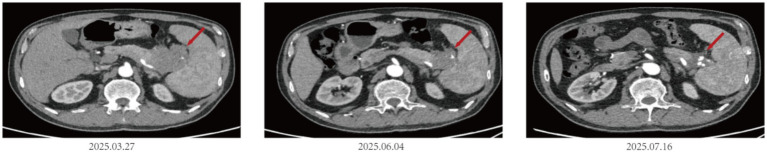
CT imaging of the pancreas during the course of combination therapy for small cell lung cancer with brain metastases.

Concurrent systemic evaluation demonstrated that the primary lung lesion significantly shrank from 3.1 × 2.0 cm on March 27, 2025, to approximately 1.0 cm on June 4, 2025, and remained at 1.0 cm on July 16, 2025(Figure 2). The pancreatic metastatic lesion also markedly reduced in size, from 5.7 × 5.6 cm on March 27, 2025, to 3.9 × 3.0 cm on June 4, 2025, and further decreased to 3.9 × 2.3 cm on July 16, 2025(Figure 3).

#### Tumor markers

During treatment, neuron-specific enolase (NSE), which is highly associated with small cell lung cancer, showed a continuous downward trend: from 27.32 ng/mL on March 31, 2025, to 9.25 ng/mL on July 9, 2025, and further to 8.39 ng/mL on September 3, 2025. Pro-gastrin-releasing peptide (Pro-GRP) also decreased significantly, from 1549.31 pg./mL on March 31, 2025, to 32.14 pg./mL on July 9, 2025, and measured 35.88 pg./mL on September 3, 2025, remaining within normal levels. These markers gradually normalized after treatment, indicating a deep biochemical remission.

#### Progression-free survival

The patient achieved approximately 6 months of intracranial and systemic disease control from the initiation of anlotinib combined with temozolomide on May 16, 2025.

**Figure 4:**
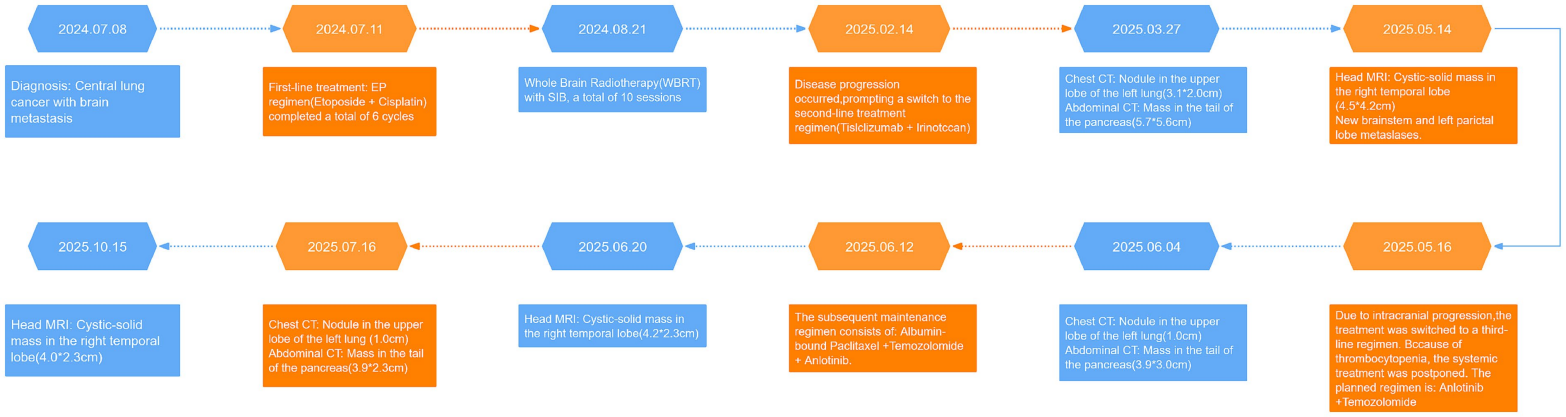
Treatment flowchart.

The treatment flowchart for this patient is shown in Figure 4.

## Discussion

The management of recurrent brain metastases after WBRT in extensive-stage SCLC is particularly challenging. Conventional cytotoxic chemotherapy demonstrates limited intracranial efficacy, largely due to inadequate penetration of the blood–brain barrier (BBB) and cumulative treatment-related toxicity (9, 10). Although immune checkpoint inhibitors have improved overall survival in extensive-stage SCLC, accumulating evidence suggests that their activity against established or recurrent brain metastases remains modest, especially in the post-WBRT setting (11). The lack of clinical benefit observed with second-line immunotherapy-based treatment in the present case further reflects this therapeutic gap.

Temozolomide, an oral alkylating agent with excellent BBB penetration, has emerged as a potential salvage option for brain metastases across multiple tumor types (12). In relapsed SCLC with brain involvement, temozolomide monotherapy has demonstrated objective response rates ranging from 12 to 25%, with a median progression-free survival of approximately 3.5 months. However, its clinical benefit remains limited when used alone, emphasizing the need for rational combination strategies to enhance intracranial and systemic disease control.

Anlotinib is a multi-target tyrosine kinase inhibitor that exerts its antitumor effects primarily through the inhibition of VEGFR2/3, FGFR1-4, and PDGFRα/*β* pathways (13–16). In the microenvironment of SCLC brain metastases, tumor cells highly express proangiogenic factors such as VEGF, driving aberrant angiogenesis, which not only supplies blood to the tumor but also increases blood–brain barrier (BBB) permeability and exacerbates peritumoral edema. Anlotinib can inhibit VEGFR2 phosphorylation, thereby blocking the downstream PI3K/Akt signaling pathway, directly suppressing the proliferation and migration of intracranial endothelial cells, reducing aberrant angiogenesis, and decreasing BBB permeability, which in turn alleviates peritumoral edema and inhibits tumor growth (17, 18).

Previous retrospective studies have demonstrated that anlotinib exhibits definite intracranial activity in patients with SCLC brain metastases, with detectable effective drug concentrations in the cerebrospinal fluid, suggesting a certain ability to penetrate the BBB. Additionally, anlotinib can reduce pericyte support of neovessels by inhibiting the PDGFR pathway, promote tumor vascular normalization, and ameliorate hypoxia in the tumor microenvironment. This may synergize with temozolomide, enhancing the cytotoxic effect of chemotherapy on intracranial lesions (19–21). In the present case, the stabilization of intracranial lesions and the reduction of NSE and Pro-GRP levels to the normal range may be a clinical manifestation of the synergistic effect between anlotinib and temozolomide, mediated by anlotinib’s antiangiogenic activity and improvement of the tumor microenvironment.

This case also carries important clinical implications. First, it supports the feasibility of an oral-based salvage regimen for heavily pretreated SCLC patients, offering convenience and manageable toxicity. Second, it highlights the potential role of antiangiogenic agents in overcoming BBB-related treatment resistance after radiotherapy. Finally, it provides a rationale for further exploration of anlotinib-based combination strategies in SCLC patients with brain metastases, particularly those with early recurrence following WBRT.

Nevertheless, several limitations must be acknowledged. First, as a single-center, single-case retrospective observation, the sample size is extremely limited, which restricts the generalizability of the findings and precludes reliable assessment of the efficacy and safety of the regimen in broader patient populations. Second, systematic biomarker analyses, including PD-L1 expression level, tumor mutational burden (TMB), and driver gene mutation status, were not performed. Therefore, the underlying molecular mechanisms of treatment response could not be investigated, and the characteristics of the potential responder population remain unclear. Third, the follow-up duration was relatively short, and long-term survival data—including long-term progression-free survival (PFS) and overall survival (OS) outcomes—are lacking, which limits the evaluation of the long-term prognostic impact of this combination regimen. Finally, the dosages and administration strategy of the three-drug combination were determined empirically, without rigorous dose-escalation or optimization studies. Consequently, the optimal dosage, treatment schedule, and dose modification strategy remain to be defined, and further exploration is needed to optimize long-term tolerability and efficacy. These limitations indicate that future prospective studies with larger sample sizes, multicenter designs, and integration of biomarker analyses and long-term follow-up data are warranted to further validate the efficacy and safety of this regimen.

## Conclusion

For patients with extensive-stage SCLC who experience early intracranial relapse after WBRT and demonstrate resistance to multiple systemic therapies, the combination of oral anlotinib and temozolomide presents a promising therapeutic strategy. This treatment plan effectively controls both intracranial and systemic disease progression, with manageable tolerability and significant prolongation of progression-free survival. Prospective, large-scale clinical studies are warranted to further define the optimal timing, dosing strategies, and predictive biomarkers associated with this treatment approach.

## Data Availability

The raw data supporting the conclusions of this article will be made available by the authors, without undue reservation.
